# An increase of serum CA-125 to two times of nadir level strongly predicts the image-identified relapse of serous ovarian cancer

**DOI:** 10.1038/s41598-024-65760-4

**Published:** 2024-07-01

**Authors:** Kaiwen Du, Qian Li, Jin Huang, David Wai Chan, Jinjin Li, Xiaoxia Chang, Hanjie Wang, Junying Tang, Qiyu Yang

**Affiliations:** 1 Department of Obstetrics and Gynecology, The First Affiliated Hospital of Chongqing Medical University, No.1 Youyi Road, Yuzhong District, Chong Qing, 400000 People’s Republic of China; 2 Department of Obstetrics and Gynecology, The Second Affiliated Hospital, The Chinese University of Hong Kong-Shenzhen, Shenzhen, People’s Republic of China; 3 Department of Obstetrics and Gynecology, The Chinese University of Hong Kong, Hong Kong SAR, People’s Republic of China; 4 School of Medicine, The Chinese University of Hong Kong-Shenzhen, Shenzhen, People’s Republic of China

**Keywords:** Cancer, Cancer imaging, Gynaecological cancer, Tumour biomarkers

## Abstract

Using 70 U/ml or 35 U/ml as CA125 routine abnormal threshold may result in omissions in the relapse detection of Ovarian cancer (OvCa). This study aimed to clarify the association between a biochemical relapse (only the elevation of CA125) and an image-identified relapse to predict the relapsed lesions better. 162 patients who achieved complete clinical response were enrolled from women diagnosed with stage I-IV serous ovarian, tubal, and peritoneal cancers from January 2013 to June 2019 at our center. The CA125 level of 2 × nadir was defined as the indicator of image-identified relapse (*P* < 0.001). Compared to CA125 level exceeding 35 U/ml, the 2 × nadir of CA125 improve the sensitivity of image-identified relapse (84.9% vs 67.4%, *P* < 0.001); the 2 × nadir value can act as an earlier warning relapse signal with a longer median time to image-identified relapse (2.7 vs. 0 months,* P* < 0.001). Of the relapsed population, there was no difference of CA125 changing trend between the neoadjuvant chemotherapy (NACT) and primary debulking surgery (PDS) group after initial treatment. Compared with 35 U/ml, CA125 reaching 2 × nadir during the follow-up process might be a more sensitive and early relapse signal in patients with serous OvCa. This criterion may help guide patients to be recommended for imaging examination to detect potential relapse in time.

## Introduction

Ovarian cancer is the second deadliest gynecological cancer, with a 5-year survival rate of 50.8%^1^. Over 80% of these women are diagnosed as serous epithelial tissue^2^. The standard treatment involves cytoreduction surgery and chemotherapy. Primary debulking surgery (PDS) followed by platinum-based chemotherapy is the preferred initial treatment for advanced-stage ovarian cancer. Neoadjuvant chemotherapy (NACT) followed by interval debulking surgery (IDS) is an alternative option for those who cannot undergo PDS. While many randomized clinical trials have shown the non-inferiority of NACT in survival outcomes^3,4^, a recent study found that women in the NACT group experienced more relapses in the pelvis or upper abdomen compared to the PDS group^5^. Regardless of the treatment option, complete resection of the tumor through surgery remains crucial in reducing the relapse rate and improving survival.

Ovarian cancer recurs in 25% of early-stage and more than 80% of advanced-stage women^6^. Effective follow-up tests are needed to detect relapse or disease progression. Screening methods include physical examination, serum tumor biomarkers, and imaging techniques. The most widely used tumor biomarker is CA125, a glycoprotein found on the surface of ovarian cancer cells since 1981. Additional maker as HE4 have been introduced in Guideline, but CA125 seems to be the most reliable marker for disease monitoring^7^. The Gynecologic Cancer InterGroup (GCIG) has already accepted the research result by Rustin that CA125 levels more than twice the upper limit of normal (2 × ULN) predict tumor relapse^8^. However, with the significant improvement of the density resolution technology in medical imaging, using the traditional cut-off value of 2 × ULN U/mL for relapse is being questioned. Studies have shown that even within the normal range(< 35 U/mL), three progressively rising CA125 at 1- to 3-month intervals^9^ or an increase of more than 5 U/mL from 3 to 6 months after treatment^10^ are associated with a higher risk of relapse. Therefore, a more sensitive cut-off point is needed.

In fact, relying solely on CA125 levels is not sufficient to confirm relapse. A rise to more than the cut-off value of CA125 several months before the evidence of image-identified relapse or symptomatic clinical relapse is called biochemical relapse. Early treatment based on elevated CA125 levels alone can cause anxiety and side effects without improving survival^11^. Excessive imaging screening would cause unnecessary anxiety and financial burden. Undoubtedly, predicting image-identified relapse based on CA125 elevation is challenging but important. Additionally, initial treatment strategies can cause CA125 profiles to be worse in those having NACT than PDS as NACT patients have more advanced disease in the real clinical world. However, the tendency of significant deviation in CA125 will disappear following treatment, as CA125 will reduce to a low level after a combination of surgery and chemotherapy. Exploring the changing trends of CA125 profiles till relapse after the two distinct treatments in the real clinical world is expected to contribute more evidence to guide clinical practice in this area.

In this study, the aim is to explore the association between biochemical relapse (elevated CA125) and image-identified relapse of ovarian cancer to improve the prediction of relapsed lesions requiring treatment. Additionally, a subgroup analysis in relapsed patients was performed to clarify the effects of different initial treatments on the relapsed outcomes at the biomedical level.

## Material and methods

### Study population

All women diagnosed with International Federation of Gynecology and Obstetrics (FIGO, 2013) stage I-IV ovarian, tubal, and peritoneal cancers from January 2013 to June 2019 at the Department of Gynecology, The First Affiliated Hospital of Chongqing Medical University, China, were reviewed. We retrospectively collected clinical data of 162 women who achieved complete clinical response (CCR) following primary treatment and met the following inclusion criteria: (1) All pathological types were serous; (2) Relapse was confirmed by imaging and was treated accordingly; (3) Without other malignancy; (4) Complete clinical data; (5) Regular follow-up; (6) CCR. Those who achieved without of CCR, received previous treatment for biochemical relapse, and had irregular follow-up were excluded.

In the current study, CCR was achieved when the criteria were met: the absence of tumor-associated clinical symptoms, the negative signs of residual tumor on the physical examination and imaging study results, and a serum CA125 level below the ULN (35 U/ml) after primary treatment. Image-identified relapse was identified with the occurrence of any new measurable lesion as revealed through imaging studies.

### Collection of clinical information of the enrolled women

(1) Women’s baseline characteristics and initial treatment: age at diagnosis, body mass index (BMI), FIGO stage, primary tumor type, CA125 level at diagnosis, the median number of total chemotherapy cycles, surgical procedures, operation time, blood loss, surgical outcome, PARP inhibitor use, and histologic grade; (2) CA125 level per treatment phase: at diagnosis, before IDS, at the end of primary therapy, at nadir during follow-up, at 2 × nadir, at the image-identified relapse, and time intervals between the key disease-progression points; (3) Women’ relapsed characteristics: imaging method, number of sites of relapse, specific sites of relapse (pelvis, upper abdomen/diaphragm, distant, lymphatic, carcinomatosis), and the extent of relapse (intra-abdominal, extra-abdominal, ascites/effusion). The censor date was March 2023.

### Follow-up

Check-ups were performed every three months for the first 2 year post-treatment, every three to six months for the third to fifth years, and annual follow-ups for the sixth year**.** The follow-up items included: gynecological examination, gynecological ultrasound, serum CA125, and other serum tumor markers. The CA125 was measured by the same assay during follow-up in each patient. The nadir CA125 levels of individual patients were the lowest biomarker value after primary therapy during follow-up, and the 2 × nadir level is two times of the lowest CA125 value after primary therapy during follow-up. The highest level of CA125 was defined as the apex of the follow-up CA125 level whose measurement can be confirmed without relapsed disease. An annual imaging check-up consisted of a chest high-resolution computerized tomography (HRCT) scan and an abdomen and pelvic contrast-enhanced computerized tomography (CT) or magnetic resonance imaging (MRI). Whole-body positron emission tomography-computed tomography (PET-CT) was performed if a distant metastasis was highly suspected. The Response Evaluation Criteria in Solid Tumors (RECIST) and the World Health Organization (WHO) criteria were used to assess tumor therapy response and clinical relapse.

### Statistical analyses

Continuous parameters were presented as the median with IQR (inter-quarter range) and analyzed by the Mann–Whitney test. The rank categorical variables were described as numbers (percentages) and analyzed with the Wilcoxon rank sum test. 35 U/ml and 2 × nadir of CA125 level were set as the cut-off values of relapse in all included patients, and comparisons of correlated sensitivity and specificity were made using the size of Youden’s index. Next, 35 U/ml and 2 × nadir of CA125 level were set as the key points in relapsed patients, and comparisons of correlated proportions were made using the McNemar test. The IBM SPSS Statistics 26.0 software was used for statistical analysis. Violin plots and ROC curve were created with GraphPad Prism 9. *P* values < 0.05 were considered statistically significant.

### Ethical approval

This study was approved by the Ethical Committee of The First Affiliated Hospital of Chongqing Medical University (protocol code 2022-K404 and date of approval 1 September 2022) and conducted in accordance with the Declaration of Helsinki. Each patient had given written informed consent at the time of treatment for the future use of their clinical data. This study is compliant with all institutional and national guidelines and regulations for human subjects research.

## Results

### Baseline characteristics of the present study population

**Figure 1:**
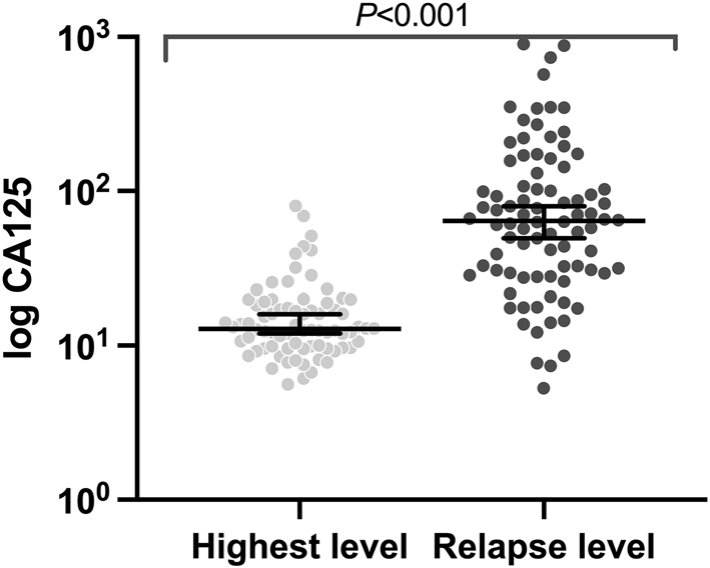
Relapse levels of CA-125 in patients who developed image-identified relapse of serous ovarian, tubal, and peritoneal cancers (median, 64.0 U/ml; IQR, 29.4 U/ml to 134.1 U/ml) were significantly higher than the highest CA-125 level recorded during follow-up in patients who did not develop relapsed disease (median, 12.8 U/ml; IQR, 9.8 U/ml to 18.8 U/ml; *P* < 0.001).

The clinical baseline characteristics of the patients are given in Table 1. Of the 162 patients who achieved CCR following primary treatment included in the present study, 86 (53.1%) patients who were diagnosed with imaging-identified relapsed disease were defined as the relapse group, and 76 (46.9%) patients who were confirmed without relapsed disease as the relapse-free group. Most patients’ (143, 88.3%) tumors originated from the ovary. 52 (68.4%) and 75 (87.2%) patients had high-grade primary tumors in the relapse-free and relapse group, respectively (*P* = 0.006). Approximately 69.7% were classified in FIGO stage II/III in the relapse-free group and 75.6% were classified in FIGO stage III/IV in the relapse groups, respectively (*P* < 0.001). Likewise, the CA125 level at diagnosis in the relapse group was significantly higher than in the relapse-free group (*P* = 0.026). In primary treatment, both the proportion of NACT (52.3% vs 35.5%, *P* = 0.032) and distant metastases resection (15.1% vs 2.6%, *P* = 0.006) in the relapse group was significantly higher than that of the relapse-free group. In the surgical outcome, no residual tumors were achieved in more than half of the women in the relapse-free and relapse groups, respectively (71.1% vs 53.3%, *P* = 0.008). The two groups had similar baseline features, including age, BMI, tumor origin, Median number of chemotherapy cycles, pelvic and para-aortic lymphadenectomy. The median CA-125 level at image-identified relapse (64.0 U/ml; IQR, 29.4 U/ml to 134.1 U/ml) was significantly higher than that of the median pre-relapse highest CA-125 level (12.8 U/ml; IQR, 9.8 U/ml to 18.8 U/ml; *P* < 0.001; Fig. 1).

**Table 1:** Baseline characteristics of the present study population (162 women with ovarian, tubal, and peritoneal cancer).

	All (N = 162)	Relapse-free (N = 76)	Relapse (N = 86)	*P*-Value
Age at diagnosis, years	52 (47–61)	52 (47–61)	52.5 (47–60)	0.997
BMI, kg/m^2^	22.8 (21–24.8)	22.7 (20.9–24.7)	22.8 (21.2–25)	0.900
FIGO stage				< 0.001*
I	13 (8.0%)	12 (15.8%)	1 (1.2%)	
II	45 (27.8%)	25 (32.9%)	20 (23.3%)	
III	67 (41.4%)	28 (36.8%)	39 (45.3%)	
IV	37 (22.8%)	11 (14.5%)	26 (30.2%)	
Tumor origin				0.974
Ovary	143 (88.3%)	67 (88.2%)	76 (88.4%)	
Fallopian tube	12 (7.4%)	7 (9.2%)	5 (5.8%)	
Peritoneum	7 (4.3%)	2 (2.6%)	5 (5.8%)	
CA125 at diagnosis, U/mL				0.026*
≤ 500	54 (33.3%)	32 (42.1%)	22 (25.6%)	
≤ 1000	45 (27.8%)	20 (26.3%)	25 (29.1%)	
≤ 2000	23 (14.2%)	9 (11.8%)	14 (16.3%)	
> 2000	40 (24.7%)	15 (19.7%)	25 (29.1%)	
Primary treatment				0.032*
NACT + IDS	72 (44.4%)	27 (35.5%)	45 (52.3%)	
PDS + ACT	90 (55.6%)	49 (64.5%)	41 (47.7%)	
Median number of chemotherapy cycles	7	7.5	7	0.912
Surgical procedures				
Pelvic lymphadenectomy	65 (40.1%)	32 (42.1%)	33 (38.4%)	0.630
Para-aortic lymphadenectomy	26 (16.0%)	12 (15.8%)	14 (16.3%)	0.933
Abdominal organ resection	13 (8.0%)	8 (10.5%)	5 (5.8%)	0.272
Distant metastases resection	15 (9.3%)	2 (2.6%)	13 (15.1%)	0.006*
Operation time, min	188 (150–236)	188.5 (150–227.5)	188 (150–238.5)	0.930
Blood loss, mL	200 (100–400)	200 (100–400)	200 (100–410)	0.571
Surgical outcome				0.008*
RT = 0	100 (61.7%)	54 (71.1%)	46 (53.5%)	
RT < 1 cm	35 (21.6%)	16 (21.1%)	19 (22.1%)	
RT ≥ 1 cm	27 (16.7%)	6 (7.9%)	21 (24.4%)	
Histologic grade				0.006*
High-grade serous	127 (78.4%)	52 (68.4%)	75 (87.2%)	
Low-grade serous	15 (9.3%)	12 (15.8%)	3 (3.5%)	
Serous not specified	20 (12.3%)	12 (15.8%)	8 (9.3%)	

All data are no. of women (%) or median number (IQR).

Abbreviations: BMI, body mass index; FIGO, International Federation of Gynecology and Obstetrics; NACT, neoadjuvant chemotherapy; IDS, interval debulking surgery; PDS, primary debulking surgery; ACT, adjuvant chemotherapy; RT, residual tumor.

*Statistically significant variables and statistically significant results are indicated in bold.

### Prediction of imaging-identified relapse using follow-up CA-125 level

**Figure 2:**
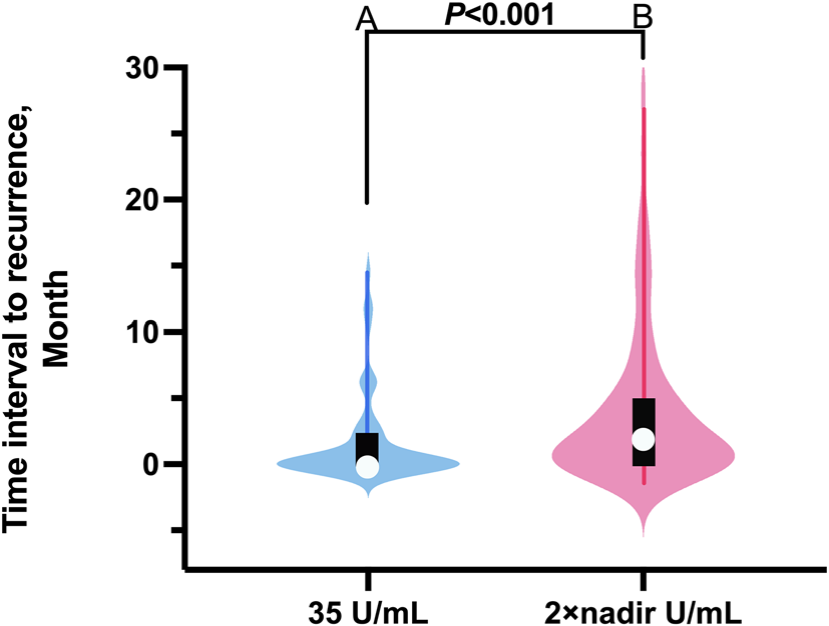
The time interval characteristics of the 35 U/m group and the 2 × nadir group in 86 relapsed women. If the CA125 concentration at the image-identified relapse exceeded 35 U/mL, patients were defined as the 35 U/ml group; If the CA125 concentration at the image-identified relapse exceeded its 2 × nadir, patients were defined as the 2 × nadir group. (**A**) 35 U/ml group: violin plot of time intervals (median, 0 months; IQR, 0 months to 2.19 months) between firstly reaching 35 U/mL of CA125 and image-identified relapse in fifty-eight women (58, 67.4%) ; (**B**) 2 × nadir group: violin plot of time intervals (median, 2.67 months; IQR, 0.85 months to 6.25 months) between firstly reaching 2 × nadire U/mL of CA125 and image-identified relapse in seventy-three women (73, 84.9%). Inside each violin plot is a box plot showing the quartile, median, and range distribution. There are significant differences between red violin and blue violin distributions in each plot.

To get closer to the clinical reality, we evaluated two CA125 cut-off values that have significant predictive value for relapse: ULN (35 U/ml) and the 2 × nadir level. Of the 162 patients who achieved CCR following primary treatment, 64 (39.5%) had an increased CA125 level to 35 U/ml and 87 (53.7%) had an increased CA125 level to 2 × nadir during follow-up. When the cut-off value of CA125 was 35 U/mL, the sensitivity and specificity for predicting clinical relapse were 67.4% and 92.1% respectively; When the cut-off value of CA125 reached 2 × nadir, the sensitivity and specificity for predicting clinical relapse were 84.9% and 81.6% respectively. The difference was statistically significant, the Youden’s index of the 2 × nadir U/ml was higher than 35 U/ml (0.665 vs 0.595, Table 2). Of the 86 patients with imaging-identified relapse, 58 (67.4%) showed a CA125 level reaching 35 U/ml, while 73 (84.9%) showed a CA125 level getting 2 × nadir before relapse. The difference was statistically significant (*P* < 0.001, Table 3). Of 86 women experiencing relapse, 17(19.8%) whose follow-up CA125 reached its nadir × 2 level without reaching 35 U/ml could be identified as high-risk populations of omissions in clinical practice (Table 3). Moreover, as shown in Fig. 2, if the CA125 concentration at the image-identified relapse exceeded 35 U/mL, patients were defined as the 35 U/ml group; If the CA125 concentration at the image-identified relapse exceeded its 2 × nadir, patients were defined as the 2 × nadir group. The median time interval from CA125 reaching 35 U/mL to image-identified relapse was 0 months (IQR, 0 to 2.19 months), while that from CA125 reaching 2 × nadir to image-identified relapse was 2.67 months (IQR, 0.85 to 6.25 months), with a statistically significant difference (*P* < 0.001, Fig. 2).

**Table 2:** Image-identified relapse prediction using the CA125 cut-off values of 35 U/ml and 2 × nadir U/ml in 162 women with ovarian, tubal, and peritoneal cancer.

	Image-identified relapse		All
	Yes (86, 53.1%)	No (76, 46.9%)	(162)
CA125 level reached 35 U/ml, N (%)			
Yes	58 (67.4%)*	6 (7.9%)	64 (39.5%)
No	28 (32.6%)	70 (92.1%)*	98 (60.5%)
CA125 level reached its nadir × 2 level, N (%)			
Yes	73 (84.9%)*	14 (18.4%)	87 (53.7%)
No	13 (15.1%)	62 (81.6%)*	75 (46.3%)

*The results of sensitivity and specificity are indicated in bold.

CA125 nadir value is the lowest biomarker value after primary therapy during follow-up.

Youden’s index = sensitivity + specificity-1. The Youden’s index of the 2 × nadir U/ml as cut-off value was higher than 35 U/ml (0.665 vs 0.595).

**Table 3:** The classification of 86 relapsed women based on different CA125 cutoff values.

	CA125 level reached its nadir × 2 level, N (%)		All
	Yes	No	
CA125 level reached 35 U/ml, N (%)			
Yes	56 (65.1%)	2 (2.3%)	58 (67.4%) *
No	17 (19.8%)	11 (12.8%)	28 (32.6%)
All	73 (84.9%) *	13 (15.1%)	86 (100%)

*Statistically significant variables and statistically significant results are indicated in bold.

CA125 nadir value is the lowest biomarker value after primary therapy during follow-up.

### Baseline characteristics and relapsed characteristics of relapsed patients

Among the 86 women with image-identified relapse, 41 (47.7%) women undergoing PDS followed by ACT were defined as the PDS group, and 45 (52.3%) women undergoing NACT followed by IDS were defined as the NACT group, for the initial treatment. The baseline and relapsed characteristics of 86 relapsed women are listed in Table 4. At initial diagnosis, 68.3% of the women in the PDS arm had a serum CA125 level lower than 1000 U/mL, while 57.8% of the women in the NACT arm showed a CA125 over 1000 U/mL (*P* = 0.003). Approximately 95.5% and 53.7% were classified in FIGO stage III/IV in the NACT and PDS groups, respectively (*P* < 0.001). Likewise, the median number of chemotherapy cycles in the PDS arm is six compared to eight in the NACT arm (*P* = 0.019). The two groups with relapse had similar baseline features, including age, BMI, and primary tumor type. High-grade serous histology was observed in nearly 90% of women in each group. Both complete and optimal surgeries were achieved in more than half of the women (53.7% vs. 53.3% in the PDS and NACT groups, respectively). No differences were observed in the surgical parameters between the two groups with relapsed disease.

**Table 4:** Baseline and relapsed characteristics of 86 women with relapsed ovarian, tubal, and peritoneal cancer.

	ALL(N = 86)	PDS (N = 41)	NACT (N = 45)	*P*-Value
Age at diagnosis, years	52.5 (47–60.3)	51 (45–55)	54 (48–63)	0.060
BMI, kg/m^2^	22.8 (21.1–24.9)	23.1 (21.6–25.2)	22.7 (20.8–25)	0.489
FIGO stage				< 0.001*
I	1 (1.2%)	1 (2.4%)	0	
II	20 (23.3%)	18 (43.9%)	2 (4.4%)	
III	39 (45.3%)	15 (36.6%)	24 (53.3%)	
IV	26 (30.2%)	7 (17.1%)	19 (42.2%)	
Tumor origin				0.194
Ovary	76 (88.4%)	38 (92.7%)	38 (84.4%)	
Fallopian tube	5 (5.8%)	3 (7.3%)	2 (4.4%)	
Peritoneum	5 (5.8%)	0	5 (11.1%)	
CA125 at diagnosis, U/mL				0.003*
≤ 500	22 (25.6%)	13 (31.7%)	9 (20%)	
≤ 1000	25 (29.1%)	15 (36.6%)	10 (22.2%)	
≤ 2000	14 (16.3%)	6 (14.6%)	8 (17.8%)	
> 2000	25 (29.1%)	7 (17.1%)	18 (40%)	
Median number of chemotherapy cycles	7	6	8	0.019*
Surgical procedures				
Pelvic lymphadenectomy	32 (37.2%)	16 (39%)	16 (35.6%)	0.940
Para-aortic lymphadenectomy	14 (16.3%)	9 (22%)	5 (11.1%)	0.204
Abdominal organ resection	5 (5.8%)	1 (2.4%)	4 (8.9%)	0.204
Distant metastases resection	12 (14.1%)	5 (12.2%)	7 (15.9%)	0.625
Operation time, min	188 (150–238.5)	185 (151–233.8)	190 (137.5–275)	0.612
Blood loss, mL	200 (100–405)	200 (100–400)	300 (100–460)	0.302
Surgical outcome				0.460
RT = 0	46 (53.5%)	22 (53.7%)	24 (53.3%)	
RT < 1 cm	19 (22.1%)	5 (12.2%)	14 (31.1%)	
RT ≥ 1 cm	21 (24.4%)	14 (34.1%)	7 (15.6%)	
PARP inhibitor use	35 (40.7%)	15 (36.6%)	20 (44.4%)	0.459
Histologic grade				0.899
High-grade serous	75 (87.2%)	36 (87.8%)	39 (86.7%)	
Low-grade serous	3 (3.5%)	1 (2.4%)	2 (4.4%)	
Serous not specified	8 (9.3%)	4 (9.8%)	4 (8.9%)	
Imaging method of relapse				
PET-CT	35 (40.7%)	17 (41.5%)	18 (40%)	0.105
MRI	31 (36.1%)	14 (34.2%)	17 (37.8%)	0.726
CT	14 (16.3%)	7 (17.1%)	7 (15.6%)	0.849
Puncture under color ultrasound	14 (16.3%)	6 (14.6%)	8 (17.8%)	0.693
Number of sites of relapse				0.764
1	28 (32.6%)	14 (34.1%)	14 (31.1%)	
2 +	58 (67.4%)	27 (65.9%)	31 (68.9%)	
Sites of relapse				
Pelvis	54 (62.8%)	22 (53.7%)	32 (71.1%)	0.094
Upper abdomen/diaphragm	10 (11.6%)	4 (9.8%)	6 (13.3%)	0.741
Distant (brain/chest/liver/spleen parenchyma)	28 (32.6%)	7 (17.1%)	21 (46.7%)	0.003*
Lymphatic	31 (36%)	15 (36.6%)	16 (35.6%)	0.921
Carcinomatosis	27 (31.4%)	10 (24.4%)	17 (37.8%)	0.182
Intra-/Extra-abdominal				0.629
Intra-abdominal	71 (82.6%)	33 (80.5%)	38 (84.4%)	
Extra-abdominal	15 (17.4%)	8 (19.5%)	7 (15.6%)	
Ascites/effusion	6 (7%)	2 (4.9%)	4 (8.9%)	0.678

All data are no. of women (%) or median number (IQR).

Abbreviations: PDS, primary debulking surgery; NACT, neoadjuvant chemotherapy; BMI, body mass index; FIGO, International Federation of Gynecology and Obstetrics; RT, residual tumor; PARP, poly (ADP-ribose) polymerase, PET-CT, positron emission tomography-computed tomography; MRI, magnetic resonance imaging; CT, computed tomography.

*Statistically significant variables and statistically significant results are indicated in bold.

As shown in Table 4, most women were detected to have multiple relapsed lesions by the examination of PET-CT and MRI. The pelvis was the most common site of relapse (62.8%) and was detected with a higher percentage in the NACT group than in the PDS group (71.1% vs. 53.7%, *P* = 0.094). The incidence of distant relapses (brain/chest/liver/spleen parenchyma) in the NACT group was significantly higher than in the PDS group (46.7% vs. 17.1%, *P* = 0.003). An intra-abdominal relapse was far more common than an extra-abdominal one in all women (82.6% vs. 17.4%), with the same trend in both groups.

### CA125 values and time intervals between the key disease-progression points of relapsed patients

Serum CA125 values at various time points and time intervals were presented in Table 5. For all the relapsed women, the median CA125 level at diagnosis was 929.9 U/mL, IQR from 495.8 to 2256.6 U/mL. Relapsed women in the NACT group showed a higher CA125 than those in the PDS group at diagnosis (1523.6 vs. 663.1 U/mL, *P* = 0.003). After initial treatment, the median CA125 value of 86 relapsed women decreased to nearly 10 U/mL, IQR from 7 to 13.8 U/mL. During follow-up, the median CA125 level at nadir was 7.8 U/mL in 86 relapsed women, and there was no difference between the two groups (7.6 vs. 8.8 U/mL, respectively). Subsequently, the disease progressed until imaging-relapsed evidence was detected. At this period, the median CA125 level in the PDS group (60.5 U/mL) was slightly higher than that in the NACT group (54.2 U/mL), but there was no significant difference between the two groups with relapsed disease. In the PDS group, it took 7.5 months from surgery to the CA125 level reaching the nadir and then 11.6 months from the nadir level to image-identified relapse. The NACT group took 6.6 months from surgery to the CA125 nadir level and 9.6 months from the nadir level to the image-identified relapse. Although the PDS group is slightly longer than the NACT group in each timespan, there was still no statistical difference between the two groups.

**Table 5:** Serum CA125 concentrations at various time points and time intervals between the key disease-progression points of 86 relapsed women.

	All (N = 86)	PDS (N = 41)	NACT (N = 45)	*P*-Value
CA125, U/mL				
At diagnosis	929.9 (495.8–2256.6)	663.1 (295.7–1647.6)	1523.6 (628.3–3005.7)	0.003*
Pre-IDS			44.55 (25–155.1)	
At the end of primary therapy	10.35 (7–13.8)	9.9 (6.9–12.2)	10.2 (6.9–14.5)	0.815
Nadir	7.8 (6.5–11.2)	7.6 (6.6–10.8)	8.8 (6.5–12.15)	0.558
2 × nadir	20.5 (14.1–36.4)	16.27 (13.9–28.9)	22.6 (14.7–38.7)	0.190
At the image-identified relapse	64 (29.4–134.1)	60.5 (28.2–91.2)	54.2 (32.2–102.8)	0.263
The time intervals of CA125 change, month				
From surgery to nadir	7.3 (6–10.7)	7.5 (6.3–12.5)	6.6 (4.7–9.1)	0.126
From the end of treatment to nadir	3.4 (1.5–6.8)	2.5 (1.2–8.2)	3.4 (1.5–5.5)	0.649
From the end of treatment to relapse	15.6 (8.6–22.8)	17.3 (8.1–25.9)	14.8 (8–20.3)	0.265
From nadir to relapse	10.7 (5.3–14.7)	11.6 (5.5–16.2)	9.6 (5.5–14.7)	0.705
From nadir to 2 × nadir	6.77 (3.6–9.4)	8 (3.2–12.3)	5.8 (3.6–8.5)	0.294
From 2 × nadir to relapse^a^	2.67 (0.9–6.3)	2.6 (0.8–6)	2.8 (0.6–6.3)	0.591
From 35 U/mL to relapse^b^	0 (0–2.2)	0 (0–0.8)	0 (0–2.4)	0.269

All data are median number (IQR).

Abbreviations: IDS, interval debulking surgery.

CA125 nadir value is the lowest biomarker value after primary therapy during follow-up.

*Statistically significant variables and statistically significant results are indicated in bold.

^a^Only 73 women analyzed the time interval from 2 × nadir U/mL when CA125 first appeared to relapse due to 13 women undergoing relapse with the CA125 value below 2 × nadir U/mL.

^b^Only 58 women analyzed the time interval from 35 U/mL when CA125 first appeared to relapse due to 28 women undergoing relapse with the CA125 value below 35 U/mL.

## Discussion

The 5-year and 12-year survival rates of relapsed ovarian cancer were less than 30% and 5%, respectively^12^. Monitoring relapse is a key aspect of the overall management of women with EOC. With CA125 levels frequently rising several months before image-identified relapse^11^, determining the imaging examination time based on the fluctuation of CA125 values could alleviate anxiety and reduce the economic burden for women with asymptomatic relapse by avoiding multiple negative imaging examinations. Therefore, defining the CA125 cut-off that signifies the need for diagnostic imaging and therapy to relapse is critical to ensure effective management.

In 1996, RUSTIN et al. posited that the criterion for tumor progression in patients whose CA125 levels fell to within the normal range is a doubling of CA125 level from the upper limit of normal (2 × ULN)^8^. In 2001, they confirmed that doubling of CA-125 from its nadir level has now been shown to accurately define progression in patients with persistently elevated levels over the normal range^13^. Based on the research results of RUSTIN’s team, the GCIG accepted the CA125 criteria into the Response Criteria in Solid Tumor (RECIST) to predict ovarian cancer relapse. The criterion of EOC relapse has remained relatively stable over the past 20 years; in comparison, the density resolution technology in medical imaging has improved significantly, and more timely and accurate detection of smaller lesions was found. Using a CA125 level of 2 × ULN (70 U/ml) as part of the criterion remains uncertain. The difference between RUSTIN’s study and our study is that we investigated whether 35 U/ml and 2 × nadir of CA125 level can increase the accuracy of progression instead of traditional 2 × ULN of CA125. Compared to the population of RUSTIN’s study, in our study, we selected relatively stable patients whose CA125 decreased to normal (< 35 U/ml) and showed complete clinical response after primary treatment with regular follow-up. The CCR patients who were detected with an increased CA125 level of 2 × ULN during follow-up were only 37 (22.8%), which was significantly lower than that of 35 U/ml and 2 × nadir.

However, using 35 U/ml to replace 2 × ULN as the CA125 abnormal threshold for all CCR women in general also may result in omissions or delays in relapse detection. In this study, by using the sensitivity and specificity of the 2 × nadir U/ml and 35 U/ml to compare the size of Youden’s index**,** we found that CA125 reaching 2 × nadir during the follow-up process might be a more sensitive and early relapse signal in women having completed initial treatments for serous ovarian cancer. Moreover, more than half of the women (17, 60.71%) who never reached the upper limit of the normal range of 35 U/mL at the time of relapse showed a CA125 level over 2 × nadir. The combination of 2 × nadir and 35 U/mL predicted outcomes better than 35 U/mL alone, which could significantly improve the detection rate of relapse.

Many studies have redefined the value of CA125 in predicting relapse. A rise of 5 U/mL or 10 U/mL from the nadir CA125 level has been associated with relapse in women who had a complete response to therapy^14^. Wang et al. proposed that the CA125 level of 1.68 × nadir was defined as the indicator of relapsed disease^15^. In a French multicenter study, nadir CA125 values below 20 kU/L were associated with more prolonged Overall Survival (OS) and Disease-free Survival (DFS) (*P* < 0.0001)^16^. However, these studies aimed to reveal the association between CA125 elevation and the ultimate relapse without proposing a specific follow-up strategy. In the only randomized trial ever performed of CA125 monitoring after the initial treatment, patients who only experienced the increase of CA125 were assigned to early treatment, and patients with clinical or symptomatic relapse were assigned to delayed treatment^11^. The results showed no evidence of the survival benefit of early treatment. This finding challenged the widespread belief by denying routine recommend of CA125 follow-up and the early treatment of relapse based on a raised CA125 concentration alone. Actually, monitoring CA125 has been a routine in conventional follow-up, and effective but slightly expensive imaging examinations, such as computed tomography (CT), magnetic resonance imaging (MRI), positron emission tomography/computed tomography (PET/CT), were used as an additional check when CA125 is abnormal^17,18^. Therefore, our study wants to analyze and investigate the correlation between early and delayed treatment groups in the study mentioned above, whether the raised CA125 concentration alone can predict the clinical relapse of the delayed treatment group. It is essential but challenging for asymptomatic women to determine the optimal imaging examination timing based on the fluctuation of CA125. Some gynecologic oncologists advocate a wait-and-see approach, but the duration of the waiting period and the relapse surveillance are unclear^19^. Asymptomatic women waiting for symptoms may suffer from anxiety, leading to decreased quality of life and unnecessary imaging examinations^11^. Despite the SGO and NCCN guidelines recommending that imaging only be used when clinically indicated, more than 75% of ovarian cancer women were still receiving routine surveillance for non-indication imaging^20,21^. Hence, a clear cut-off of the CA125 level to start the diagnostic imaging was required. According to our results, when the CA125 level reaches 2.095 × nadir or 35 U/mL, an imaging examination at this follow-up visit or the subsequent follow-up would be recommended. More specifically, in clinical practice, imaging examinations should be performed once the CA125 level exceeds 35 U/mL or within three months for women whose CA125 level is consistently lower than 35 U/mL but reaches 2 × nadir.

In the study of Wang et al., comparing with a CA125 level > 1.68 × nadir at relapse, women with CA125 level ≤ 1.68 × nadir at relapse can extend the overall and progression-free survival durations^15^. In the present study, the sensitivity attained using a CA125 level of 2 × nadir was relatively high (84.9%). However, our findings did not reveal a relationship between the CA125 levels at image-identified relapse and overall survival. The possible reason was that not every asymptomatic woman start treatment immediately after observing image-identified relapse. Given the selection bias inherent to retrospective studies, attempts to translate the above-mentioned favorable outcome to all CCR OvCa women should be approached with caution. Our study aimed not only to predict relapse, but also to clarify the time point to start image examination and appropriate therapy according to the CA125 levels. Based on the current results, our team would further verify the improvement effect of intervention at 2 × nadir CA125 value on survival outcomes by performing prospective trials.

Several differences were noticed between the relapse-free and relapse groups, including those in the FIGO stage, CA125 at diagnosis, primary treatment, surgical procedures, surgical outcome, and histologic grade. The higher clinical stage, CA125 level, and histologic grade frequently represented a sign of a more serious malignant condition and far-spread range of the disease, which tends to result in extending the application of neoadjuvant chemotherapy and debulking surgery. Besides, several differences were noticed between the NACT and PDS groups in the relapsed patients, including those in the FIGO stage, CA125 at diagnosis, the number of chemotherapy cycles, and the incidence of distant metastasis. These differences might be related to the severity of the disease itself in the two groups of women. Gynecologic oncologists would like to use platinum-based chemotherapy several times in advance to reduce the tumor size of women with advanced OC, aiming to achieve optimal surgery^3,4,22,23^.

Focused on relapse disease in our study, the tendency of significant deviation in CA125 disappeared following treatment, as CA125 decreased to nearly 10 U/mL, IQR from 7 to 13.8 U/mL. It is not what we expected that the changing trends of follow-up CA125 profiles till relapse after the two distinct treatments in the real clinical world was still no statistical difference. Additionally, there is evidence that most EOC and primary peritoneal cancers originated from the umbrella end of the fallopian tube. The standard treatment for these three types of cancers is almost identical to clinical guidelines. Therefore, five women with fallopian tube cancer and five women with peritoneal cancer in this study were not excluded. Limitations of this study include its retrospective and unicentric nature and the relatively small sample size. Another drawback is that we mainly focused on CA125 without considering the predictive function of other tumor biomarkers^24^.

## Conclusion

To sum up, our results showed that the CA125 level of 2 × nadir indicates the need to initiate imaging examination within three months to detect relapsed serous ovarian, tubal, and peritoneal cancers. As the combination of 2 × nadir and 35 U/mL predicted outcomes better than either alone, it may be a practical and low-cost method for oncologists to manage women with serous OvCa.

## Data Availability

The datasets analyzed during the current study are not publicly available due to privacy and ethical restrictions but are available from the corresponding author on reasonable request.

## Abbreviations

CA125: Cancer antigen 125
OvCa: Ovarian cancer
EOC: Epithelial ovarian cancer
NACT: Neoadjuvant chemotherapy
IDS: Interval debulking surgery
PDS: Primary debulking surgery
FIGO: International federation of gynecology and obstetrics
RT: Residual tumor

## References

[1] Title of Cancer Stat Facts: Ovarian Cancer. Available from: https://seer.cancer.gov/statfacts/html/ovary.html. Accessed on August 1, 2023.

[2] Lheureux S, Braunstein M, Oza AM (2019). Epithelial ovarian cancer: Evolution of management in the era of precision medicine. CA. Cancer J. Clin..

[3] Kehoe S (2015). Primary chemotherapy versus primary surgery for newly diagnosed advanced ovarian cancer (CHORUS): an open-label, randomised, controlled, non-inferiority trial. The Lancet.

[4] Vergote I, Amant F, Ehlen T (2010). Neoadjuvant chemotherapy or primary surgery in stage IIIC or IV ovarian cancer. N. Engl. J. Med..

[5] Mitsopoulos V, Innamaa A, Lippiatt J, Collins S, Biliatis I (2022). Differences in patterns of recurrence between primary and interval debulking surgery for advanced ovarian cancer. Anticancer Res..

[6] Salani R (2011). Posttreatment surveillance and diagnosis of recurrence in women with gynecologic malignancies: Society of gynecologic oncologists recommendations. Am. J. Obstet. Gynecol..

[7] Ferraro S (2018). Serum human epididymis protein 4 vs. carbohydrate antigen 125 in ovarian cancer follow-up. Clin. Biochem..

[8] Rustin GJS, Nelstrop AE, Tuxen MK, Lambert HE (1996). Defining progression of ovarian carcinoma during follow-up according to CA 125: A north thames ovary group study. Ann. Oncol..

[9] Wilder JL (2003). Clinical implications of a rising serum CA-125 within the normal range in patients with epithelial ovarian cancer: a preliminary investigation☆. Gynecol. Oncol..

[10] Piatek S (2020). Rising serum CA-125 levels within the normal range is strongly associated recurrence risk and survival of ovarian cancer. J. Ovarian Res..

[11] Rustin GJ (2010). Early versus delayed treatment of relapsed ovarian cancer (MRC OV05/EORTC 55955): a randomised trial. The Lancet.

[12] Charkhchi P (2020). CA125 and ovarian cancer: A comprehensive review. Cancers.

[13] Rustin GJ, Marples M, Nelstrop AE, Mahmoudi M, Meyer T (2001). Use of CA-125 to define progression of ovarian cancer in patients with persistently elevated levels. J. Clin. Oncol. Off. J. Am. Soc. Clin. Oncol..

[14] Gadducci A, Cosio S (2009). Surveillance of patients after initial treatment of ovarian cancer. Crit. Rev. Oncol. Hematol..

[15] Wang F (2013). CA-125–indicated asymptomatic relapse confers survival benefit to ovarian cancer patients who underwent secondary cytoreduction surgery. J. Ovarian Res..

[16] Riedinger JM (2006). CA 125 half-life and CA 125 nadir during induction chemotherapy are independent predictors of epithelial ovarian cancer outcome: Results of a French multicentric study. Ann. Oncol..

[17] Salani R, Khanna N, Frimer M, Bristow RE, Chen L (2017). An update on post-treatment surveillance and diagnosis of recurrence in women with gynecologic malignancies: Society of Gynecologic Oncology (SGO) recommendations. Gynecol. Oncol..

[18] Tanner EJ (2010). Surveillance for the detection of recurrent ovarian cancer: Survival impact or lead-time bias?. Gynecol. Oncol..

[19] Fleming L (2001). Playing the waiting game. The asymptomatic patient with recurrent ovarian cancer detected only by rising Ca125 levels. Scott. Med. J..

[20] Rimel BJ (2015). Improving quality and decreasing cost in gynecologic oncology care. Society of gynecologic oncology recommendations for clinical practice. Gynecol. Oncol..

[21] Esselen KM (2016). Use of CA-125 tests and computed tomographic scans for surveillance in ovarian cancer. JAMA Oncol..

[22] Fagotti A (2020). Randomized trial of primary debulking surgery versus neoadjuvant chemotherapy for advanced epithelial ovarian cancer (SCORPION-NCT01461850). Int. J. Gynecol. Cancer.

[23] Onda T (2020). Comparison of survival between primary debulking surgery and neoadjuvant chemotherapy for stage III/IV ovarian, tubal and peritoneal cancers in phase III randomised trial. Eur. J. Cancer.

[24] Vallius T (2017). Postoperative human epididymis protein 4 predicts primary therapy outcome in advanced epithelial ovarian cancer. Tumor Biol..

